# Supplementary material for: Collaboration for Developing and Sustaining Community Dementia-Friendly Initiatives: A Realist Evaluation
Source: Int J Environ Res Public Health. 2023 Feb 23;20(5):4006. doi: 10.3390/ijerph20054006 (PMC10001691; doi:10.3390/ijerph20054006)
Supplement: Supplementary file 1 [file ijerph-20-04006-s001.zip › ijerph-2116755-supplementary-file1-v3.pdf]

Interview guide focus group

Belonging to Mentality; a study of success factors of dementia-friendly initiatives and communities.

Participants: Members of the CoP and other stakeholders who have been involved in the development and implementation of 2-3 dementia-friendly initiatives. These members are specifically invited by researcher and facilitator.

Allocated time: 90-max 120 minutes.

Location: preference by the CoP.

Method; see below

Practical organization in advance:

- Book a quiet room in consultation with the CoP. Provide coffee and tea.
- Send the participants a week in advance the information letter about the focus group and invite them.
- Bring audio recording equipment.
- Make sure there is a flipchart in the room or bring your own laptop and beamer.
- Bring the interview guide, paper, pen and business card.
- Provide sufficient informed consent forms.
- Create a list of participants with space for names and signatures.
- Ensure that logic model from the reflection tool is available to focus group participants, either with a presentation or bring your own.
- It also depends on the space, mutual distance and the possibility of reading along; Instead of a presentation or flipchart or slide, you can also make prints for everyone on A4 and hand them out. (You may have already used this option in the first focus group, in which case you can reuse it)

Preparation: material & content

- Provide an overview of actions from the logs and minutes, for example on a timeline to give an overview of what has been done (actions). Consider in advance which actions were most appropriate to the theory, which will support you during the focus group.
- Go through the reflections of the facilitator and researcher and note the most important signals. Formulate with the facilitator some probing questions that could be asked.

- Divide the role between the researcher and facilitator. The researcher is the one who leads the focus group and asks the questions, probing and summarizing. The facilitator takes notes and shares them when requested by the researcher, for example when providing summaries.

Regarding role facilitator:

- It is very likely that cooperation aspects will be discussed continuously, so make separate notes for the discussion of the relevant theory.
- Draw the interviewer's attention when questions based on signals are relevant, agree with each other what the most pleasant working method is.
- Discuss examples from the other pilot municipalities with the other researcher; it may be desirable in the conversation to mention examples from other contexts in order to provoke / sharpen answers / provoke discussion.
- Bring sticky notes; if you want to give people the opportunity to think about an answer or if you want to collect several answers from everyone or specific people (whom you then specifically ask).

Preparation: technical conversation

- The conversation focuses on testing the theory of collaboration; when enough information has been gathered, complete the topic and move on to subsequent questions. Also important in the context of time management.
- Probing is an important interview technique for concretizing answers or identifying underlying motives or perspectives. Asking for examples is a way of asking: Can you give an example of that or questions such as: Can you tell us more about that? Can you explain how you see that? etc. Try to get it as clear as if you can actually ‘see’ it.
- Summarizing is a very important interview technique, you give back to the participant what you heard and whether you understood it correctly.
- Listen and see which words best resonate with the participants. For example: if the initiative is not clear (enough), use the activity or word that the participant uses for it. This facilitates the conversation. This also applies to the choice of the word 'theory' or description. Choose what best fits the understanding of the participants.
- Build in some thinking space sometimes, pause.
- When using post-its: it is up to you as a moderator to make audible what is written on the post-its, by having the participants introduce themselves (aloud) or by summarizing yourself.

Focus group:

Introductie

- Welcome all participants.
- Indicate again the aim of the focus group as stated in the information letter: reflection on cooperation in order to develop or safeguard dementia-friendly initiatives
- Indicate that you use audio recording equipment to listen back to what has been said.
- Indicate that the data will be treated confidentially and that we will keep the audio recording in a secure place.
- Request permission to use the audio recording. Ask the participants to sign the informed consent form.
- Indicate that a member check will take place. The focus group proceeds will be shared with the participants and other members of LG who can add their feedback/input. After that, the yields are determined.
- Turn on the audio recording equipment.

Introduction text (example):

Information project: the text below is optional and is also included in the information letter that participants received

Welcome everyone and thank you for being here! Today we have the first/second focus group of the project Mentality. In this project we investigate success factors of dementia-friendly initiatives. In the previous phases of the project, we conducted literature research into success factors of dementia-friendly initiatives according to people with dementia and their informal caregivers. We also visited 4 best practices- municipalities that were already making progress in the development of dementia-friendliness. We spoke to professionals from health care and welfare, municipal policy officers and volunteers who were involved in one or more dementia-friendly initiatives. We asked them questions about the success factors in the development and sustainment of dementia-friendly initiatives. One of the success factors is collaboration. This success factor is summarized in a theory or generic description in the reflection tool. Characteristic of these descriptions is that they describe causality; it therefore indicates how a certain outcome is achieved in the collaboration.

In the coming year/six months we will work together on developing dementia-friendly initiatives, in which the current description of the collaboration will be described even better and more accurately with the help of your experiences and also the experiences of people with dementia and their carers. After the project has ended, we can also support other municipalities that want to become dementia-friendly.

Focus group introduction tekst

Today's meeting focuses on data collection. Today/Six months ago we have/had the first focus group, today is the first/second focus group. In the past period, we have worked hard on the (further) development of dementia-friendly initiatives. With this we also learn a lot about what is involved in the creation and sustainment of the initiatives in case 2-4. With your experiences, we discuss to what extent the theory/description from the reflection tool about collaboration is correct and/or needs to be adapted up when it concerns cases 2-4.

The focus group consists of several parts; first we go through the actions and then we reflect on your experiences based on the theory/description of collaboration.

Introduction: Overview of the actions (30 minutes max)

As the start and possible warm-up of this focus group, we reflect on the actions that have been taken in the past six months to develop and sustain dementia-friendly initiatives.

Questions	Background
Based on the logs and minutes we have made an overview of actions taken by you, take a look at it. Do you recognize 'your' actions?<br>*Do you have additions and if so, which ones?*	Introducing and completing actions<br><br>Ensure that there is consensus in this group regarding the actions taken. There will be another member check, so additions are welcome.<br><br>Give participants the opportunity to respond to each other and invite participants to do so. Example questions: I hear you say … and I hear you say …, can you explain to each other how you think the situation works?
Which actions did you find more and less easy/appealing to undertake?	Introduction, 'warming up' by thinking about actions.
Good to see that people with dementia and carers were involved in the actions; what has that achieved for you? How do you look back on it? / Do you think it is worth repeating? Why/why not? What would you like to change and why?<br><br>*OR*	Focus on the role of people with dementia and their carers in the development of dementia-friendly initiatives.<br><br>Continue to ask about motivations, underlying reasons. Possible input for follow-up

Have people with dementia and carers also been involved in these actions? Why/why not? How did that go? How do you reflect on it? / Do you think it is worth repeating? Why/why not? What would you like to change and why?	
What influence has Corona had on your actions? For example, what has not been done, or what has been done? What have you changed or modified?	

Part 2: Interview questions about the development and sustainability of dementia-friendly initiatives (max 60 minutes)

Six months ago you discussed with each other that the development and sustainability of dementia-friendly initiatives was one of the ambitions. Today we are going to use your experiences to test and refine previously developed theories about it. To this end, we look back at the collaboration of the past period and also use previously collected information such as observations, reflections and minutes of the meetings.

Questions	Background
Which actions were most important for the collaboration? Can you explain why? Does this also have a link with the characteristics of the neighborhood in [name of municipality]	Contextual aspects<br>Go back to the overview of the actions. Present what you know and ask for confirmation. helped by the facilitator who listened and wrote along, and ask you for any additions.
What characterizes your collaboration? In the CoP and with other parties involved?<br><br>*Collaborations have also been examined from other studies and people were involved in this based on personal connection with dementia, there was transparency about the budget and decisions were made together. Do you recognize that in your own collaboration? Which ones and which ones not?*	Contextual aspects<br><br>Possibly supported by signals and observations from facilitator and researcher

What do you think are the success factors of the collaboration? Did you expect that?/ Is that characteristic of [name of municipality] or otherwise?<br><br>*Success factors are also known from previous research, for example, people keep each other informed, within the collaboration they do what suits everyone's interests and there is clarity and guidance in the collaboration. Do you recognize that or is it different here? Why/why not?/ Is that characteristic of [name of municipality] or otherwise?*	If possible, name the link/similarity between self-named - and the success factors of the theories
What are the /outcomes of your collaboration? / How has collaboration improved over the past six months?<br>*Were there any unexpected/unforeseen outcomes? Or so-called 'extra-catch'?*	If possible, name the link/similarity between self-named - and the success factors of the theories
In a previous study, outcomes of collaboration were defined as 1) people who remain committed to a dementia-friendly initiative 2) satisfaction, pleasure and purposefulness during the collaboration 3) people who want to take initiative. Do you recognize that (also?) Can you explain similarities/differences? Why do you think that is?	If possible, name the link/similarity between self-named - and the success factors of the theories
How do you think these outcomes were achieved? /What changes have your actions brought about that enabled your outcomes to be achieved?<br><br>*Previous research (in other municipalities) showed that outcomes were achieved because people felt personally important and connected to the realization of the dementia-friendly initiative. How do you see*	Questions about mechanism responses<br>Attention for facilitator for a summary of actions and outcomes,<br>Asking questions about what changed 'in the head and in the heart', which was decisive for the outcomes.

that? Do you have similar experiences? Can you cite examples of that?<br><br>If not:<br><br>*Can you tell us a little more about other experiences you've had? How do you look back on that now? / What ideas did you get?*	Mechanisms: ask about reflections; what changed ‘in the head/in the heart' that was decisive for the outcomes.<br><br>It can also be negative experiences, also very important.

Completion

- Thank the participants for their attendance and valuable contribution. Make it a so-called 'moment of success' by handing out a gift. Please also bring this with you to the next meeting of the CoP where a member check will take place.
- Indicate that a member check will take place. The outcome of the focus group will be submitted to those present in the CoP for approval. After that, the findings are determined.
- Indicate how we process the data and what we do with the data.
- Indicate briefly what the continuation of the project will look like in terms of research.
- Close the meeting.
