# Supplementary material for: Collaboration for Developing and Sustaining Community Dementia-Friendly Initiatives: A Realist Evaluation
Source: Int J Environ Res Public Health. 2023 Feb 23;20(5):4006. doi: 10.3390/ijerph20054006 (PMC10001691; doi:10.3390/ijerph20054006)
Supplement: Supplementary file 1 [file ijerph-20-04006-s001.zip › ijerph-2116755-supplementary-file2-v3.pdf]

Interview guide exit interviews

Belonging to Mentality; a study of success factors of dementia-friendly initiatives and communities.

Purpose of the interview: to gain insight into mechanisms and factors within Case 1 that influenced case 1 not to form a learning community for the development of dementia-friendly initiatives and community.

Interviewer: Independent researchers who are not involved with Case 1.

Duration: 30-45 minutes

With whom: Relevant stakeholders of the municipality who can reflect on the activities undertaken in the context of the project.

Practical organization:

- Make an online appointment
- Reserve and bring audio recording equipment
- Provide 2 informed consent forms per participant (one for interviewer, one for participant)
- Interview guide, paper and pen for possible. take notes if necessary

Preparation:

Structure of the interview: the interview consists of two parts. Part 1 looks back at the motivation to participate in the project and the expectations regarding outcomes for the municipality. Part 2 asks about the participant's perspective on the activities undertaken, choices made and the outcome of terminating participation.

Introduction

- Explain to the participant:
    - Name the purpose of the interview.

  - Aim for a 'conversation', no wrong or right answers.
  - Use of audio recording equipment
  - Measures regarding privacy, anonymous processing of the data, secure storage of the data.
- Request permission to use audio recording and ask participant to sign Informed consent form
- Turn on the audio recording equipment
- Before each interview, record: date of the interview and code of the participant.

START INTERVIEW

Introduction

Thank you for taking the time to do this interview today. At the end of March, in consultation with the municipality, it was decided that Case 1 would withdraw from the Mentality project.

Unfortunately, it was not possible to form a learning community. We regret this. Through this interview we would like to learn from the experiences of participants in Case 1. We would like to gain more insight into the ambitions in Case 1, expectations you had with regard to participation in the Mentality project, what went well, what did not and what made you decide to stop.

Part 1: Dementia friendliness ambition, participation in Mentality and motivation (10-15 minutes)

Questions	Background information/inquiries
Was there an ambition to become dementia-friendly? If so, how did it come about? Was there an urgency in case 1?	

What signals did you get? Were there needs, ideas?<br><br>*What was the reason for Case 1 to participate in the study? What was your motivation for participating?*<br><br>*What appealed to you and why?*<br><br>*What expectations did you have at the start? What could/should the project deliver to the municipality of Case 1? What did you envision?*	How did you come into contact with the study?<br><br>How do you reflect on this?<br><br>Previous experiences with other projects<br><br>Experience with method of collaboration in this study and vision on this.

Part 2: Reflection on the activities undertaken, choices made and the outcome to end participation (30-45 minutes)

Questions	Background information/inquiries
How do you look back on the process to arrive at dementia-friendly initiatives?<br><br>What do you think of the actions/ steps taken, why? What do you think of the result?<br><br>What do you think of this approach?<br><br>Did the collaboration/development within the municipality go as you expected? Or as it normally goes in your municipality?	Present an overview of the steps taken (broadly speaking, moments of decision).

What is/isn't?<br><br>What was an important event/meeting/moment in this process? And why?<br><br>How do you see your own role in the whole and in the project? Was this different than expected? What did you like/dislike about this?<br><br>What are you satisfied/unsatisfied with? Why?<br><br>After 6 months of trying, it was not possible to form a learning community. This was a prerequisite for the project to continue.<br><br>What do you think of the idea of a learning community to create collaboration to achieve dementia friendliness?<br><br>What do you think was/were the main reason(s) for this failure?<br><br>Some municipalities take control during the development, while others take a more wait-and-see approach.<br><br>How do you view the direction of the municipality in the development of a	What is characteristic of the collaboration in Case 1?<br><br>Perhaps a connection with motivation/ideas from part 1.<br><br>For example, a difference in development at a municipality such as X, where the municipality has worked proactively and with a plan from the start.

dementia-friendly community? Where do you think the direction should be?	Does the composition of the municipality play a role in this?
Besides management, are there other factors that you think are important in Case 1? For example ownership?	Connection to Part 1 of the interview, Experiences from the past. Did this happened more often?<br>Can you name an example?
Are there characteristics of Case 1 that influence the collaboration and the development of dementia-friendly initiatives?	How do you think it will succeed in Case 1 and why?
How does the municipality view collaboration, connections between partners within the municipality, the role of a network and the role of the municipality in this?	
How does the municipality view sustainability and embedding such projects in the municipality?<br>How does this work in Case 1?	
Which factors in Case 1 offers faith in to creating a dementia-friendly community?	

Are there any topics that we have not discussed that you would like to mention to complete the picture?	
