# Supplementary material for: Collaboration for Developing and Sustaining Community Dementia-Friendly Initiatives: A Realist Evaluation
Source: Int J Environ Res Public Health. 2023 Feb 23;20(5):4006. doi: 10.3390/ijerph20054006 (PMC10001691; doi:10.3390/ijerph20054006)
Supplement: Supplementary file 1 [file ijerph-20-04006-s001.zip › ijerph-2116755-supplementary-file3-v3.pdf]

Additional file Patterns: outlines after cross case synthesis, based on cross synthesis of CMOc of cases 1-4.

Each table represents an intermediate outcome, supported by a selection of quotes.

**1. Feeling the need for DFC**					
	**Cases**	**Context**	**Mechanisms-resource**	**Mechanism-response**	**Outcomes**

	Case 1-4	having and disseminating expertise in dementia<br><br>(not) having a heart for the cause<br><br>(not) mixing DFI with other activities in the district<br><br>(not) having a clear picture or story of the target group for which the DFI is intended<br><br>(not) focusing on development of DFIs (but on the internal process)<br><br>(not) having one's own story of target group or meaning<br><br>(not) keeping the perspective of the person with dementia and the caregiver central	awareness of diversity/that there is dementia in the neighbourhood<br><br>(not) being open to the target group/affinity to guiding/supporting the target group<br><br>shared wishes/identity<br><br>(not) feeling a connection with the target group and its needs<br><br>PWD and MZ who are / feel personally addressed	feeling more familiar with the target group<br><br>realizing what knowledge and skills are required<br><br>feeling capable<br><br>feeling involved with the idea/people<br><br>(not) feeling the urgency/necessity for DFIs<br><br>(not) understanding the impact of DFIs<br><br>PWD and MZ who feel heard / taken seriously	Recognizing the need for a DFC

Quotes:

- We don't really talk much about dementia, or about people with dementia.
  SPM: No that's true.
  SP1: Or about experiences and such.
  SPV: I think we are missing – that's what we were talking about at the time – the connection with it. Like we said, we need to bring someone along sometime. Take someone along once, where comes... But those are not things we have been busy with... Because we are continuously working on: what are the roles, who does what. We have not actually reached that depth, in my opinion (Case 3-T2)
- But I really just like it. That's where my heart lies, doing this. So, this is double for me. This will actually be my job, but I also find it really fun to do. I also recognizable what you say. It's actually half work for me, half my own ambition, drive, the desire to improve things a bit in Wijchen.

Yes, so you sometimes chose to make time for it, even when you actually didn't have it?
SPV: Yes
SP1: What made it valuable to you to make time for it?
SPV: Well, exactly what the working group itself has also stated, or in the learning community: you just see that there are people who want to. Those who are ready and willing to help (Case 3-T2)

- Yes, and additionally chance is also a factor. If you are present in the neighbourhood, you sometimes just happen to run into people. For example, here is a 90-year-old man who comes around here and says 'hey, I hear there are people around who have problems using technology' for example. I would like to give a technology course here..... There are those people who will not proactively say 'hey I want to contribute to it' but when you are talking to them it is so easy to make that offer. (Case 4-T1)
- SPV: But I believe in this, that this [collaboration for DFIs with people with dementia and carers involved] could work, yes.
  SP1: So what exactly is what you believe in?
  SPV: That there will be more room for people with dementia and their carers to lead a meaningful and pleasant life in the neighbourhood and to be involved in that process, that is actually what it is about. And what we should always keep in mind, that's the goal it's all about (Case 2-T2)
- the degree of concreteness of whether someone can immediately visualize this in their own environment. What does it mean for me? Yes, then perhaps people are more inclined to say: yes, I will participate in that, than that it will immediately get so big that it will be a bit abstract. (M-T1)
- In making the decision, well, there is a bit of politics behind it, to choose the elderly boards of a few cores first, but then you also do not choose other organizations that also deal with the elderly. They are very concentrated on their own cores, and therefore you can ask yourself to what extent they feel in touch with the other side of the Meuse and with each individual senior. (Case 1-T1)
- Ambitious. I actually think yes, of course. I notice that to a larger extent in the past six months. But when I just talk to you, also in the Connection working group, I see a lot of great big plans, great examples. So, I have the feeling that Nijmegen East is really ambitious when it comes to dementia-friendliness. (Case 2-T1)
- Regardless of one's background, you have to have something to do with it. SPV: Yes, but I don't think you would join otherwise. I don't think we need to spend a lot of thought or attention, maybe not a lot of time, on emphasizing the importance of a dementia-friendly neighbourhood, because we do agree on that. We all have that vision. We don't have to start a discussion among ourselves about who are we talking about and what we want. (Case 2-T1)

**2. Clarity about input from partners**					
	**Cases**	**Context**	**Mechanisms-resource**	**Mechanism-response**	**Outcomes**

	Case 2-4	Top-down approach in management and communication (municipalities-policy-PR folders)<br><br>supply-driven approach-little insight into needs<br><br>informing about the approach<br><br>physical meeting with involved partners<br><br>positive response to LG members' ambitions<br><br>open communication with each other	insight into the matter (complexity of DF)<br><br>insight into target group)<br><br>(lack of) recognition of the need for DFI<br><br>confirmation/appreciation for commitment<br><br>making an effort to understand each other	(not) gaining insight into the complexity of dementia-DF<br><br>reflection (asking yourself about the approach),<br><br>not feeling seen/heard by others<br><br>motivation/commitment to other LG members/network partners<br><br>(not) feeling taken seriously<br><br>feeling safe together<br><br>being sensitive to each other-loyal	familiarity with the approach<br><br>being able to pick up personal input/role<br><br>making concrete and clear actions/ agreements<br><br>stagnation - no goal-oriented approach<br><br>smooth dealing with each other in cooperation

Quotes about one's own input:

- It is difficult to make a connection with the municipality, but also if you are dealing with a person who should be working on something then it is yes, I am not working on it or I do not understand it or I will ask my colleague who it's there...yes, it gets put off a bit...and it must be for a good reason, but then it stagnates a lot. Yes, NW has replaced that. It is indeed difficult to know how to make that connection again. (Case 4-T1)
- If you take the time together to also go over those small steps that you have taken or those small new collaborations that you have researched, if you share them with each other, then you can easily find new energy or new collaboration or you can start sparring with each other about taking it one step further.
  And then I think we are doing very well. For example, regarding the pieces that have appeared in the local newspaper or the interviews with the residents themselves. There is always room and time in this learning community to really discuss it. But how often does that actually happen? And yes, I see real added value

in that. If you want to work well together or if you want to strengthen the cooperation with each other, if you share your own successes or what your cooperation has already achieved, then that gives you energy. Then it is not my successes, but the successes of the collaboration, for example, to take the time for that (Case 2-T2)

**3. Mutual support**					
	**Cases**	**Context**	**Mechanisms-resource**	**Mechanism-response**	**Outcomes**
	Case 2-3	diversity in professional and volunteer partners, diverse roles and expertise,<br><br>Getting to know each other in terms of expertise and backgrounds<br><br>Informal atmosphere<br><br>insight into existing qualities and possibilities - getting familiar with each other	Coordination<br><br>Expanding network - knowing who is who<br><br>Connecting with each other, shorter lines of communication<br><br>Each one has a unique contribution<br><br>Wider orientation/support<br><br>Regularly getting various incentives/information from various partners.	feeling mutually connected<br><br>feeling of looking after each other<br><br>feeling supported<br><br>broader picture on the subject and the neighbourhood<br><br>starting to find it (more) important - thinking about it more often	Supported/mutual support/ambition

Quotes about support for ambitions:

- That is also about sensitivity in relationships, insight into relationships. And I don't mean at all if you say, if you would say very bluntly: okay Margreet can, she's from the municipality. You can see that. And then you can do that and that. But how does that compare? I mean, I just say it like it is. But then there is also the

matter: B also has the same interests. The goal. A nice person. So, everything just falls in place. I don't want to explain it too bluntly, B, because I value you very highly. It's about: I need B.

SPV: Yes, and I need S. and I need you all.

SPV: Yes, as you say, I also have to think of A who is at the 1st informal care cafe with his wife. You are there, you know. That's so important, we always need each other, but he is also just a very nice person. We have the same goal. So, what I mean is that... It's about sensitivity. How to achieve things with others (Case 3-T2)

- Yes, affinity, but also information about what it means or what things are going on.
  And I think it is important that in such a working group you can also compare different experiences. I can imagine that they (people with dementia and their carers) may have a different image or have different goals or have different experiences. What are we talking about then? Well, I believe you do that with people from different perspectives and angles, so different areas. I do think we could reinforce that by broadening that perspective even more, but we've talked about that before. But I do think that we are sitting down with the right partners to do that. I wouldn't know what an alternative could be, so to speak, unless you're going to do it all on your own, but I don't believe in that. I think that you should indeed get it done from the collaboration and different expertise, yes (Case 2-T1)
- It's really nice to have someone to spar with in any case, and to share your experiences, and maybe even have someone do something if you think: hey, I can use that there in a situation. And I also like to be with the group; it gives me energy to continue, yes especially, even if they are only small successes, but especially to hold on to those successes and everyone's enthusiasm to continue. Yes
  SP1: And so that enthusiasm does something to you too?
  SPV: Yes, exactly, yes. That also gives me energy to spend my time here again, yes. (Case 2-T2)
- If you take the time together to also go over those small steps that you have taken or those small new collaborations, if you share them with each other, then you can easily find new energy or new collaboration or you can start sparring with each other about taking it one step further. (Case 2-T2)

**4. Interconnection**					
	**Cases**	**Context**	**Mechanisms-resource**	**Mechanism-response**	**Outcomes**

	Case 1-3	Use of media, word of mouth, physical meetings to spread the idea of DF<br><br>Involved organizations (also towards each other), being aware of what everyone is doing, commitment to each other and a common goal.<br><br>Flexible attitude of the municipality with regard to rules<br><br>Well-known partners from the neighbourhood who specifically invite LG for their commitment/input because of their approachability and place in the neighbourhood<br><br>Having ears and eyes for each other's experiences in the development of DFI<br><br>connect with the interests of supporters (organizations)<br><br>Open communication about progress of DFI development, commitment, hours and people	being/feeling personally concerned<br><br>shared wishes/identity<br><br>appreciating each other's ideas and efforts.<br><br>Recognition of the stories and needs<br><br>Create space to come up with your own input/interest<br><br>equal input from (future) LG members-staying in the conversation<br><br>Mutual interest of organizations	understanding the importance of DFI/DFC-<br><br>understanding the importance of their efforts, feeling responsible/commitment towards the people involved. co-ownership<br><br>feeling the trust of others (LG members and organizations) being active<br><br>Freedom-sense of direction<br><br>Increasing the enthusiasm, awareness and involvement in the ambition of DF<br><br>feeling (less) supported<br><br>feeling (less) taken seriously	Interconnection.<br><br>giving space for your own story and interest. Reciprocity

Quotes:

- It is not that I receive hours to spend on this. I take hours for it. Luciver gives me space. Because they want to be proactive and want me to invest in Wijchen, in the area. And in the network I should say. But you have to justify why it is necessary every time. And then the advantage is that a health insurer attaches great value to cooperation in the environment. So, it yields something, yes it’s true, it ultimately also yields something for the organization, that you can make contacts more easily. So, yes, it is double so to speak (Case 3-T2)
- And especially the volunteers, who um, they form a large part of it, maybe one third, they eventually came through word of mouth so that in in, because she says oh, I know someone who might like that and actually that’s where we are now, at least then we have enough volunteers. (M-T1)
- In the choice, well, there was a bit of political choice behind it, to choose the elderly boards of a few cores first, but then you also do not choose other organizations that also deal with the elderly. They are very concentrated on their own cores, and you can ask yourself to what extent they feel in touch with the other side of the Meuse and with each individual senior. (Case 1-T1)
- If that is Case 1, a much larger municipality, much, yes, and however very diverse municipalities too, I think that on some fronts, the social cohesion may also be less………….. ...and then you notice that that was not conducive to forming the community that you developed a connection, together, a sense of responsibility because they were all very different people from different backgrounds, for a manager, say, professionals and people from or from specific advisory groups. They didn't have much in common beforehand, so that didn't work well (Case 1-T1)
- That the professionals were so much more involved. And we had to beg, as it were. We were able to have conversations with the professionals, but a lot came out like oh, then you have to do this and you have to talk to them. But a group in which professionals also contribute ideas and develop, was…

  SP1: Suddenly all the doors opened.

  SPV: Suddenly all doors opened, yes. We were two of those ladies who were allowed to do their thing, but who above all did not have to take up any time. This is a citizens' initiative. PV: But then you see that you don't actually get the inputs you need. (Case 2-T1)
- we just have to take those first steps first, and I already talked about that with A., the next step is simply to write to the supermarkets, for example, or to organize something bigger. Yes. Then we just have to look. But first those first steps, and they just haven't been taken yet. Well, that might be an option, because that's one more thing I wanted to say. There are therefore also individual residents who have responded to the piece in the district newspaper.

  We had not foreseen that when we made the story with the three of us. But that is also a target group, just not a neighbourhood resident who has a neighbour who has dementia, but just interested neighbourhood residents who also just want to know on the street, what do I do if I come across someone? (Case 2-T2)
- Ambitious. I actually think yes, of course I notice a lot more in the past six months, but when I just talk to you, also in the Connection working group, I see a lot of great big plans, great examples. So, I have the feeling that Nijmegen East is really ambitious when it comes to dementia-friendliness. There is a lot of will. And yes, I mean, the flip side is when it comes down to who is going to do what? Yes, then it is more difficult. So sometimes when you're ambitious on a big level, it's hard to take small steps. I see those two sides expressed in the word ambitious. (Case 2-T2)

5. Facilitation					
	Cases	Context	Mechanisms-resource	Mechanism-response	Outcomes
	Case 1-4	Steering role of municipality coordinating person, use flexible regulations to support LG and DFI<br><br>Diversity in partners: professionals and volunteers, diverse roles and expertise. new partners with their own input.<br><br>Be familiar with the why/purpose of DF/DFI<br><br>Actions in front of and behind the scenes and being aware of them and understanding why. Encourage big plans<br><br>External: availability of education. Clarity of what can / cannot be done with regard to corona measures, manpower for guidance	Facilitation in person-coordinator and rules<br><br>Behind the scenes from organizations. Experience certainty, structure, clarity<br><br>knowing what is going on and what is needed<br><br>having a clear idea of what is needed<br><br>have an overview (as opposed to experiencing an abstract process)<br><br>commitment to each other and goal versus losing grip.	Taking on your own role and connecting to ambition-ownership experience (as opposed to feeling overwhelmed)<br><br>feeling calm and supported. feeling of (in)security/(dis)trust<br><br>feeling motivated<br><br>feel connected to each other	Taking and securing your own role<br><br>Support and security from behind the scenes

Quotes:

- I just had very short lines of communication with the people at the informal care café. I knew you wanted something. I knew S. from back in the day. We can read and write together. In a manner of. Also with others. And then you just see, you text each other, you call each other, and within 5 minutes you have short lines of communication with Margreet. It's also about having faith that things will work out. Also in terms of finances. Because I also had to wait and see whether, it is also an arrangement that financial piece. It's not all that exciting. But it also has to do with trust. you had an ambition. But that ambition was also just manageable, let's put it that way (Case 3-T2)
- That it is also very good with just his professional background. I am a professional volunteer myself, so to speak. But I think it is very important that real professionals also play a role in such a club. The professionalism and experience that those professionals have. This gives me the opportunity to consult them immediately when in doubt. Yes, but those professionals have a very important role to, yes that sounds a bit crazy, but to be able to be voluntary.
  SPM: Yes, but as a volunteer there are some aspects that you don't have at your disposal. And those are often cognitive aspects. Which you just don't know and aren't familiar with. And then it's very nice that you have that support (Case 3-T2)
- So, it is highly dependent on the person. It's nice if the people get on well with each other, but a lot of work ends up with those volunteers... and you are also vulnerable, if there are certain people who are pulling all the weight, so to speak. If such a person drops out or gets another job or something, then you have a chance that it will collapse a bit. Yes, you are really very vulnerable. As long as it functions that way, it is of course great, but yes there are some risks involved (Case 4-T2)
- ...yes and I also have the same feeling in this project that he's the one who keeps reiterating those lines with other people. Like oh that's what you need it for or that also right with him that he's also a bit like the one who makes the connections… like a spider in the web, yes that probably also makes a difference because he also regularly stimulates it. Making sure that what we are now gaining is also safeguarded and securing those connections with them ...(Case 2-T1)
- Yes, and that it consists of different parts in which everyone takes decisive actions. We can indeed organize various initiatives to promote participation as you say XX. And as YY urges, something has to be done about that, everyone should know a little bit about dementia or dealing with dementia, and then we go for it. And ZZ than confirms: yes, we can get to that outdoor environment and so we do that. So, we are all advocates for a part that you need to put in to get the picture complete. (Case 4-T2)
- But then I do find that the conclusion for me is that it is also more quickly bound and dependent on people and whether that matches people with each other and whether they exchange things from the different neighbourhoods? But it is also vulnerable because a lot is always the same person (Case 4-T1)
- I must say that a lot of time has gone into the preparation. That was of course also due to all kinds of restriction issues, but I did not expect that it would take so much time, even for certain people who joined the learning community last time, then Mrs. M. also said something about that. That it might also be something to think about: if you delegate it, then it will all be volunteers who take it upon themselves of course. Then you are somewhat dependent on the efforts and motivation of the volunteers, because otherwise nothing can come of it (Case 4-T2)
- Somewhat looser, especially from the official organization, not clinging to all kinds of rules and things like that that ultimately do not benefit the citizen. So that you often get in the way of a lot of spontaneity. That can also be done differently (Case 1-T1).

- They are again about money or they know better what the policy is in a municipality or something, I have no idea at all. So I always find that a welcome addition. And I also think it's nice, just like Yvonne, that they, yes, even if he is not always there, but they are involved and that he can often give something just that little bit extra.
  SP1: What does that mean to you, the fact that she shows that commitment, in any way?
  SPV: That it is supported by more than just the club here, that it… Look, it is also a hot item for the municipality and yes, I don't know, it gives a feeling of support. (Case 2-T1)
- And so with the few hours that I have, I do try to address issues that exist, I mean from dementia-friendly neighbourhood is one, but there are many other initiatives that also take up those few hours. And I hardly ever talk about hours, I just do that, but it is not the case that there is room to tackle this so intensively. And I think if you want to put this right, that means that you need hours for that, so really focussed hours to at least make a statement about this. It just feels very twisted from the side of the municipality, that you think well, this is just very important, but it is difficult to deploy extra resources and professional strength on this. Because the budgets are already fairly set, both at [organisation X], at [organisations Y], [organisations Z], those are the most important large parties with which we as a municipality make agreements for the resources they receive. So, I just wanted to say that. I also find that difficult. Yes. (Case 2-T2)
- Yes, there is also a lot behind the scenes. And also what Y. does at the municipality and there are more people who are all pushing these kinds of things behind the scenes. Gosh, it might be so complicated that the method isn't as inclusive as we might like. SPV: I don't think we're there yet, yes, yes. SPV: Yes, that you must have committed volunteers who know their stuff. while we all want it to be as inclusive as possible, somehow it's not that easy. SPV: No, that is certainly not easy. (Case 2-T1)
- That stability in those people is then missing, and the question is whether it will quiet down again for a while because of the weather, and the circumstances, because it is often older people who are going to do it and I also hear a lot of fuss that they finding it difficult. What can be done now? What can't be done with corona? And that part is really about the fact that it is also partially dependent on the availability of people. (Case 2-T2)

**6. Taking initiative**					
	**Cases**	**Context**	**Mechanisms-resource**	**Mechanism-response**	**Outcomes**

	Case 1-4	Clarity of DFI<br><br>DFI known to LG, nationally or in their own context<br><br>Clarity about conditions for DFI-concrete actions<br><br>Various expertise and organizations among the organizers and implementers (including LG members)<br><br>Short lines of communication<br><br>Connecting to the concrete questions and needs.<br><br>Not too big/small budget<br><br>Control over facilities (locations, deployment) and financing.	Existing working methods<br><br>Overview and concrete ideas about organization and implementation of DFI<br><br>Clear expectations of role and commitment. About one's own role/clear positions<br><br>Clear agreements<br><br>Resources and guidance with regard to organization and implementation	Enthusiasm<br><br>Feeling familiar with DFI<br><br>Feeling responsibility<br><br>a sense of control<br><br>Being able to come up with own input - being able to make a connection with DFI.<br><br>Feeling supported/feeling connected	Commitments for LG or DFI<br>Getting started<br>Satisfaction and pleasure

Quotes:

- In the beginning I personally had the idea: well, let's get it done quickly. And the longer it lasted, the more disappointed I actually became; that flexibility has not arrived. And it depends on what it is. Sometimes the process may be unclear, and then you need to clarify the process again.
  No. But what is for you… Because when I listen to you, I actually hear disappointment about the slowness. That little action and result has been achieved.
  SPM: Yes. We've talked a lot, talked a lot (Case 3-T1)
- we should take care of PR and I know what, and we let the opportunities pass. And then I get a bit of the feeling of: yes damn it, if something doesn't happen soon then…..
  Yes, everyone's role, we talked about that last time. But also that you can hardly avoid agreeing on fixed tasks with each other. And one of those regular tasks is especially as far as I'm concerned: communication

And I also notice that drive we have – to cite an example: A. she told us at one point: we must have submitted a subsidy application before 15 June. (Case 3-T1)

- Because it's a somewhat different approach than what we've had up to now and I think this can yield a lot in the long run. Sometimes I really have to put in my last bit of energy. I have to be honest because it is quite a tough process and it takes quite a lot of patience. And sometimes I also think, oh yes, I also have so many other things to do. But I believe in this, that this could work, yes. (Case 2-T1)
- When is the collaboration a success? When you get along well. And that when you go home, you have a good feeling. You say: well, it was nice, it was fun. (Case 3-T2)
- Because if I compare it a bit with PR and communication, the articles and the training courses are very clearly invested in that, at least we have a very clear face with that. And, by whomever, also a very clear view of who is involved in the implementation, and that is for the, with the KWIEK route and with the connection, that is a bit, yes, a bit more difficult. Is that a bit more diffuse? Who the face of these initiatives is. (Case 2-T2)
- And I think we are making progress, because I would also like to make progress, if we look at last six months, we also learned how important it is for an initiative or an activity to be concrete. At a certain point, that was also very important for the KWIEK route. That was just a concrete thing. PR and communication include concrete things. Articles and training. (Case 2-T2)
- They had a very clear goal and the concept as a café is very concrete, it described very clear frameworks, when it is and when it is not and also with a whole plan, all steps that you have to go through and all of which must be involved. So that was real. That is going very well .... because it was so concrete, it went very well, because people know exactly what they can do, and they also knew what investment that company had lost. ....how much that being handed to you, from the afterwards was also factual and that the pace or yes, and actually was relatively low from the moment of. We explain what we are going to do until. (Case 1)
- the degree of concreteness of whether someone can immediately visualize this in their own environment. What does it mean to me then? Yes, that perhaps people are more inclined to say: yes, I will participate in that than that it immediately becomes something big, then it is a bit abstract. (Case 4-T2)
  Speaker 2: Yeah, well, yeah, that's right. Well, a few years ago a volunteer, who had a network, stood up and said: well, I want to see if I can get something off the ground here. Well, it's now the most, one of the most vibrant, well-run buddy projects, by purely taking that local and saying: hey, I heard that you that your children are out of the house, say, wouldn't you want to do something for someone in an hour a week, or something like that? Or yes. Nice someone for someone, something nice for you, eh, where you can make a difference and you would like to participate in it. So it must be very concrete (Case 1).
- and we still thought, you know what, then we give them the link so that they can go themselves first .. but of course nobody does that, so you really have to have a physical meeting where examples can also be given and the question is of course how it benefits the entrepreneurs. You can say yes I now recognize the signs, but am I also prepared to set up my shop accordingly... (Case 4-T1)
- The meetings for the informal care café are always very spontaneous, very pleasant, very nice. So that was no overload at all……Speaker 2: I recognize that. I liked the involvement; I remember very much that we had a meeting at your house. I don't know, that was very informal or something, C who took a leading role, and D who is always present. Yes, I don't know, that gave a lot of positive energy. (Case 3-T2)
- But I think that in particular we should start talking to each other about if you want to have a mixture of non-dementia and dementia people; it requires a completely different approach. Look, as a neighbourhood organization we can actually do little in the sense of support. We can facilitate and organize, but where does the support come from? Do you want to connect to that? But where do I get the support from? I think that's the essential question. Or should you just say: we reserve a morning for walk-ins, for example, for carers with people with dementia? (Case 4-T2)

7. Mutual commitment					
	**Cases**	**Context**	**Mechanisms-resource**	**Mechanism-response**	**Outcomes**
	Case 1-4	driving force that tightens and boosts connections. Leadership-Vision as connection<br><br>recognizing previous successes of neighbourhood development and involving/questioning people<br><br>send announcements to volunteers, give recognition for efforts, inform<br><br>structure in number of meetings and content,<br><br>equal input and position during LG meetings<br><br>short lines of communication between those involved (with via the app), knowing each other well	Having a point of contact<br><br>Clarity about meetings<br><br>Getting tools and ideas with regard to DFI development<br><br>being able to make your own contribution<br><br>open mutual communication	feeling calm and supported<br><br>taking initiative (the role that trust plays in that)<br><br>feeling a sense of belonging to something<br><br>feeling proud, having trust<br><br>feeling motivated<br><br>feeling connection with each other - equality<br><br>sense of security/trust among LG members	commitment to each other and the goal

Quotes:

- I find network building a very essential part and a very important part
  Yes. Yes. And the result of such a network, what does that mean for you? How does that feel?

SPM: Yes, that is very satisfying, that you also have to do it together. And you can also see that in this group: the link with professionals, with volunteers, with well-being… Then I think: oh yes. We often think more in the problem, and they think much more in the creativity and in the… That I think: oh you know, maybe… They also said: we have participated in such an initiative very often in other places. Then I think: you need that cooperation. And it's nice if at some point you have an image and people also think: oh wait, then I can call, because they probably know who can help (Case 3-T1)

- We got to know each other. We got to know people we didn't know before, and we also regularly shared things with each other. And I certainly think that working group, when I look back at that working group, we know where to find each other. (Case 4-T2)
- Yes, by just leaving it up to local things without there being any proper coordination, well, someone who has a somewhat coordinating role in that, then I think that yes is not so successful and that also seems to be what has happened now (Case 1)
- I have the idea that K. in has to do that for a few hours a week with the other tasks. While, if you look at R. [city], for example, what I get from the side-lines is that they are working very energetically to become dementia friendly or already are. But there is also a civil servant who can devote a lot of energy and time to this, and certainly also the local politicians. But I see an important role for someone who really takes charge, as one, as someone who is responsible for it. Someone who can go all the way for that. It has to spread out over all the various sections in the community. I think anyway, it has to be someone of good character. Who has the possibilities for this, has the responsibility for it, has the commitment and who can also make it a success. (Case 1)
- I think that your role is also important, because with that we also keep it on the agenda and think, oh yes, we're going to get back together and we're going to do something again. So you really do have a kind of a pulling role; these kinds of themes also fade very quickly into the issues of the day, of oh yes, we're busy with this and that. We notice that now, other agreements, yes, you know. It fades away very quickly. And I think that it is very important to keep in mind that we do give that a kind of continuity. And also actually planning appointments and not letting go, that's very important (Case 2-T1)
- You are so knowledgeable and have a background in this. And then I think: yes, that is something different from, for example, volunteers from the [organisation], who we really want to include in this because they are in contact with neighbourhood residents or, for example, with neighbourhood residents who say... You know, so in that sense you can generalize about your volunteers either. And it might be good to check in advance which roles there are or which functions within such a partnership. And who can do what to prevent people from giving up who say, I'm a doer, give me an activity and here is just a lot of chatter and thinking and vice versa you know. So that you look at, well, who could fulfil which role and what does that look like, how do they relate to each other? (Case 2-T2)
- the short lines of communication. I, again the example if you look at, for example, table tennis in [city], the association that indicates to me, we are with this, can you help us with this? Also a bit back to what L. says, being able to find each other. But that also knowing each other again, what M. indicated. Yes, you see, yes, I think it's something, that if I hadn't had the learning community, that I would have had to look for how I could have approached this or how we could have solved it. (Case 2-T2)

Recognized inequality-equality					
	**Cases**	**Context**	**Mechanisms-resource**	**Mechanism-response**	**Outcomes**
	Case 1-4	Optimum group size, possible in work groups.<br><br>Diversity in backgrounds and expertise<br><br>Attention to both group process and content of DFI<br><br>Equal space for everyone's input<br><br>Share intermediate results - indicate what progress has been made.<br><br>Sharing positive and negative experiences<br><br>Similar expectations regarding commitment to the LG or DFI.<br><br>Short lines of communication	Getting to know each other, the person and their ambition.<br><br>Other viewpoints<br><br>Recognizable structure<br><br>Overview<br><br>Balance in everyone's contributions<br><br>Positive, constructive atmosphere<br><br>Increased insights<br><br>Equal contributions to LG or DFI<br><br>Being able to reach and involve each other quickly	Feeling safe<br><br>Being taken seriously<br><br>Daring to think differently<br><br>Feeling the time is spent meaningfully<br><br>Feeling acknowledgment-feeling meaningfully engaged<br><br>Sense of equality<br><br>Feeling part of the whole	Fulfilment<br>Bringing in new ideas<br>Staying connected<br>Motivation

Quotes:

- Yes, it is also difficult to be in the learning community. Because we are quite busy as a care cafe. And D. who comes then, she does not have such good news. So how important it was that we should also support each other in this, when things go less well. We were very enthusiastic, so that often took the upper hand. But then they were like: yeah, we can't do that. And we don't know how to proceed. While it comes from both sides. And there has never been any initiative from that side either. (Case 3-T2)
- Well, for me it is getting to know each other and literally getting to know each other by face, being able to find each other, I think that is also the success of this collaboration for me. So, in that sense I do feel the benefits of just having participated. For example, the turnout at the learning community and working groups has always been quite high. I mean, even if it wasn't always easy or when it was tough, we were there. I also thought that was very nice. And even though, you know, the successes were small or not immediately apparent, we were there. That is what I think is very special about this collaboration. (Case 2-T2)
- On the other hand, as volunteers we may also be DIY people, where you want to get to a result quickly and have that result quickly on the table. And that is also one of the reasons that I joined the Movement working group, because I thought: now I have something concrete, let's get started (Case 3-T1)
- who does the communication, who ensures that this is arranged. That someone feels the responsibility of his task. And whether we discuss it with each other or whether someone is designated as: 'Gosh, do you want to take on that task? Then you ensure that it is communicated to the outside world.' And then that is something ongoing, it will return in everyone’s booklet in the coming period: okay, how is it going, what are we working on. Then it works.
  but the group is also so large that a lot of people also think: oh, that will be arranged. (Case 3-T1)
- N. co-operation has in any case clarified a number of things, but it has to do with how we treat each other. Do I feel that security? And for me in the end D. stopped, maybe because she didn't feel comfortable. But for me it made me feel safe in the group. SP1: Yes, but do you also say that, let's see if I understand you correctly, do you also say that safety is a more important condition for safeguarding, than, for example, a clear structure. Safety is perhaps the most important. And that also gives an opportunity to be clear. (Case 3-T2)
- But the big difference for me, I don't know if it's true for you too, is that I'm a doer and that we, or as far as I'm concerned, would have been a lot more into the doing mode, while now it's a bit more of a basis and viewed from all sides. More than what I had in mind, when I thought yes, I want to do something with that. So that did it for me. It’s a common theme, but there is a line in it that I think for… And also because of you, so to speak, a close-knit core group has developed there. Yes, and we do need that. (Case 2-T1)
- there must be transparency in the manpower. Who am I at the table, what will they do with what role? Yes, because in the beginning we had to fill in such a list, didn't you, where you came from, what your role was and something like that. That might have been nice if you shared that for the whole group. Even now I still have to think: who has been in that group now? In the beginning that group was very big, and a lot of people have gone, and then it just goes away completely. Then I think: yes, what was the role or function of those persons in that whole story? Yes, you are actually saying that you have to keep track of it continuously. If something changes, then you also take that with you and you must continue to have a clear picture of what everyone will contribute and what everyone can do. (Case 4-T2)
- Also, I really liked the added value of the students. They were really very actively involved. And they came with new ideas and I think without those ideas, the start-up would have been much more difficult. I am convinced of that. But also …everyone had ideas from different perspectives. And I think that also makes it a lot of fun. (Case 2-T2)
